# Editorial: Overcome Fear and Addiction by Manipulating Reconsolidation and Extinction of Emotional Memories

**DOI:** 10.3389/fnbeh.2020.613612

**Published:** 2020-11-05

**Authors:** Jianfeng Liu, Lin Lu, Devin Mueller

**Affiliations:** ^1^Department of Psychological and Brain Sciences, College of Liberal Arts, Texas A&M University, College Station, TX, United States; ^2^NHC Key Laboratory of Mental Health (Peking University), National Clinical Research Center for Mental Disorders (Peking University Sixth Hospital), Peking University Sixth Hospital, Peking University Institute of Mental Health, Beijing, China; ^3^Department of Biological Sciences, Kent State University, Kent, OH, United States

**Keywords:** emotional memory, fear, drug reward, reconsolidation, extinction

To survive in a complicated environment, we utilize outside cues to predict future outcomes, either for avoiding detrimental events such as fearful stimuli or for approaching rewards (LeDoux, 2012). These cue-associations are stored as aversive and appetitive emotional memories within the brain, allowing cue-induced retrieval of previous experience to guide our decisions. However, when these emotional memories become aberrant and maladaptive, they lead to mental disorders such as drug addiction and post-traumatic stress disorders (Torregrossa et al., 2011; Goode and Maren, 2019). After retrieval, reactivated emotional memories can undergo reconsolidation or be opposed by new inhibitory extinction learning (Nader et al., 2000a; Bouton and Moody, 2004; Lee et al., 2006). Because of the potential of implementing reconsolidation- and extinction-based approaches to interfere with maladaptive emotional memories and thus to treat related mental disorders, these two processes have attracted great attention in this field (Flavell and Lee, 2013). In the past decades, a great deal of effort has been devoted to understanding the behavioral and molecular mechanisms of reconsolidation and extinction of many types of aversive and appetitive memories; among them, fear memory and drug reward memory are the most well-studied (Nader et al., 2000b; Quirk et al., 2000; Reichelt and Lee, 2013). In this special issue, we collect five original research articles and two review articles that outline advances in the field.

Disrupting reconsolidation is considered to be a promising approach to permanently and selectively erase emotional memories. Despite growing evidence of successful modulation of reconsolidation in associative memory models, in which associations form between a conditioned stimulus (CS; such as auditory and visual cues) and an unconditioned stimulus (US, such as drug or fearful event), relatively few studies have investigated this process in instrumental memory in which an action results in a specific outcome. In this special issue, Exton-McGuinness et al. show that disruption of instrumental memory reconsolidation prevents stress-induced cocaine relapse but does not affect other forms of relapse triggered by cocaine-associated cues and cocaine itself. As instrumental learning typically involves both associative and instrumental memories, they suggest that both components should be disrupted to prevent relapse successfully. Evidence shows that a brief CS exposure procedure could be utilized to achieve the disruption of the associative memory component in the instrumental memory model. Monsey et al. show that intra-amygdala Garcinol prevents CS-induced cocaine relapse by disrupting the brief CS exposure-triggered reconsolidation through an epigenetic mechanism. Future studies should investigate the similarities and differences in the reconsolidation of associative and instrumental emotional memories at both the behavioral and molecular levels.

Most studies on reconsolidation focus on the association of a singular cue with an emotional experience. Realistically, an emotion usually becomes associated with various cues. Thus, targeting reconsolidation of a singular cue-associated memory is unlikely to disrupt all cue associations with an emotion. First, cue-triggered reconsolidation is selective to the particular cue-related associations. Second, it is impossible to know all the cues that are associated with an emotion. Recent studies propose an alternative strategy: disruption of US-induced reconsolidation to erase the reactivated US (emotional event) associated with all memories (Debiec et al., 2010; Liu et al., 2014). Here Meir Drexler et al. report that re-exposure to a weaker US prevents fear relapse, which raises an interesting question about whether the US serves as a reminder to reactivate a particular memory and then trigger reconsolidation or to weaken the original memory through a mechanism of generalization.

Another approach to interfere with emotional memories is to enhance or facilitate extinction (Quirk and Mueller, 2008), a process that has been hypothesized as the basis of “exposure therapy” in clinical settings. Yousuf et al. report that infusion of 17β-estradiol (E2) into the infralimbic cortex, a brain region proposed to form and store extinction memory, increases neuronal excitability and facilitates extinction of cocaine seeking in ovariectomized female rodents. This study suggests that E2 optimization could be a promising adjunct for enhancing extinction-based therapy in women.

Although many studies have successfully interfered with emotional memories by strategies engaging reconsolidation and extinction, controversial results have also been reported (Bos et al., 2012; Schroyens et al., 2017). Previous studies have shown that “retrieval-extinction,” a procedure implemented by inserting a constrained interval between memory retrieval and the following extinction, could disrupt fear memory and drug reward memory (Monfils et al., 2009; Xue et al., 2012). However, Struik et al. show that the “retrieval-extinction” procedure dampens the extinction of nicotine reward memory but does not affect cocaine reward memory. Notably, this study not only demonstrates discrepancies between their findings and others but also points out an important concern: drug class should be seriously considered in drug memory studies. Vaverková et al. describe one of the popular explanations for controversial findings in reconsolidation studies: boundary conditions that prevent memory retrieval from generating sufficient prediction error to destabilize memory. Importantly, Vaverková et al. also highlight a “limbo” process that separates reconsolidation and extinction, during which amnestic agents lose the ability to interfere with emotional memory. The outcomes of amnestic agents depend on the dominance of memory trace (Eisenberg et al., 2003); therefore, the “limbo” process should be paid much more attention in the future because, currently, only reconsolidation and extinction have been well studied.

Emotional memories critically guide our decisions as we rely on both appetitive and aversive memories in our daily lives. In many cases, simultaneously retrieving these two opposing memories could result in a conflict while making a decision (Kirlic et al., 2017). Bravo-Rivera and Sotres-Bayon introduce two conflict choice-based behavioral tasks, in which animals need to choose between approaching a reward and avoiding a fearful event. They summarize current knowledge on the competition between aversive and appetitive emotional memory retrieval in conflicting choice tasks. We anticipate that these choice-based emotional tasks will be widely used as preclinical models to screen novel treatments for related mental disorders and to study the phenomenon of pathological and/or excessive choices in patients suffering from related mental disorders.

In conclusion, the seven articles in this special issue represent the challenges and treatment potential of targeting reconsolidation of emotional memories and facilitating extinction learning. This special issue provides new knowledge of reconsolidation and extinction processes as well as outstanding perspectives on how emotional memory contributes to the development of related mental disorders. These contributions bring us a step closer to efficaciously interfering with maladaptive emotional memory to achieve better treatment outcomes.

## Author Contributions

JL and DM wrote the paper. All authors approved it for publication.

## Conflict of Interest

The authors declare that the research was conducted in the absence of any commercial or financial relationships that could be construed as a potential conflict of interest.
